# Cell Growth Stimulation, Cell Cycle Alternation, and Anti-Apoptosis Effects of Bovine Bone Collagen Hydrolysates Derived Peptides on MC3T3-E1 Cells Ex Vivo

**DOI:** 10.3390/molecules25102305

**Published:** 2020-05-14

**Authors:** Jianing Wang, Junli Liu, Yanchuan Guo

**Affiliations:** 1 Key Laboratory of Photochemical Conversion and Optoelectronic Materials, Technical Institute of Physics and Chemistry, Chinese Academy of Sciences, Beijing 100190, China; wangjianing@mail.ipc.ac.cn (J.W.); junliliu306@163.com (J.L.); 2 School of Chemical Sciences, University of Chinese Academy of Sciences, Yuquan Road 19A, Beijing 100049, China

**Keywords:** bovine bonecollagen hydrolysates, EGFR, MC3T3-E1 cells, apoptosis, proliferation, cell cycle distribution

## Abstract

Bovine bone collagen hydrolysates promote bone formation through regulating bone growth. However, the peptide sequences within these isolates have not been characterized. In this study, twenty-nine peptides from bovine bone collagen hydrolysates were purified and identified using nano-HPLC-MS-MS and Peak Studio analysis. HHGDQGAPGAVGPAGPRGPAGPSGPAGKDGR (Deamidation) and GPAGANGDRGEAGPAGPAGPAGPR (Deamidation) enhanced cell viability, inhibited apoptosis, and significantly altered the cell cycle of MC3T3-E1 osteoblast cells. These peptides were selected to perform molecular docking analysis to examine the mechanism underlying these bioactivities. Molecular docking analysis showed that these two peptides formed hydrophobic interactions and hydrogen bonds with epidermal growth factor receptor (EGFR) to activate the EGFR-signaling pathway, which may explain their bioactivity. These findings indicate that these and other similar peptides might be candidates for the treatment of osteoporosis.

## 1. Introduction

Osteoporosis, a common disorder, occurs as a result of unbalanced osteoblastic bone formation and osteoclastic bone resorption [1]. Osteoporosis has influenced the lives of more than 200 million people [2]. Current treatments include medicines, including fluoride [3], that promote bone formation or those, such as bisphosphonates [4], that arrest bone resorption. These pharmaceuticals may cause several side effects including nausea and abdominal pain [5]. Therefore, there is a need for safer osteoporosis treatments with fewer side effects. 

Collagen exists in skin, bones, and connective tissue and can be obtained from different sources including both marine and terrestrial animals. However, most collagen is currently obtained from pork skin and bovine bones. Moreover, bovine collagen is used in many applications in the food and cosmetics industries [6,7].

Collagen hydrolysates are usually obtained by enzymatic hydrolysis of collagen. Collagen hydrolysates could provide benefits for the skeleton [8]. Collagen hydrolysates used as food supplements improve the compositional and biodynamic characteristics of vertebrae in ovariectomized rats [9]. Moreover, collagen hydrolysates appear to be more effective than collagen. The vertebrae of ovariectomized rats fed a collagen hydrolysate withstood a load four times that withstood by those fed with collagen [9]. Therefore, in this study, collagen hydrolysates were subjected to further study.

Previously, we showed that bovine bone collagen hydrolysates can improve osteoporosis at cellular, animal, and clinical levels. Bovine bone collagen hydrolysates can promote the proliferation and differentiation of osteoblasts [10]. Additionally, bone loss in ovariectomized rats is inhibited by oral administration of bovine bone collagen hydrolysates [11]. Ovariectomized rats are common models to study the effects of collagen hydrolyates on bone loss. Moreover, clinical study results indicate that administration of collagen peptides combined with Caltrate D can promote bone formation [12]. However, the constituents contributing to the bioactivity of these peptides, as well as the underlying mechanisms responsible for their action, remain unknown. Therefore, we isolated the collagen peptide fraction, which increased proliferation and stimulation activity in osteoblasts, and used nano-HPLC-MS-MS (nano-High Performance Liquid Chromatography-Mass Spectrometry-Mass Spectrometry) and Peak Studio software to identify the bioactive peptides. Subsequently, molecular docking analysis using the identified peptides and epidermal growth factor receptor (EGFR) was performed to screen and clarify the underlying mechanisms through which these osteogenic peptides act. The peptides with greater affinity for EGFR were synthesized and their bioactivity was verified using 3-(4,5-Dimethylthiazol-2-yl)-2,5-diphenyltetrazolium bromide (MTT), CCK8(Cell Counting Kit-8), cell cycle analysis, and apoptosis assays. Finally, to identify the underlying mechanisms of action, the affinity between EGFR and EGF (epidermal growth factor) was compared to that between EGFR and the peptides with the greatest bioactivity.

## 2. Results

### 2.1. Bovine Bone Collagen Hydrolysate (BBCH) Fractionation via Ultrafiltration

**Figure 1:**
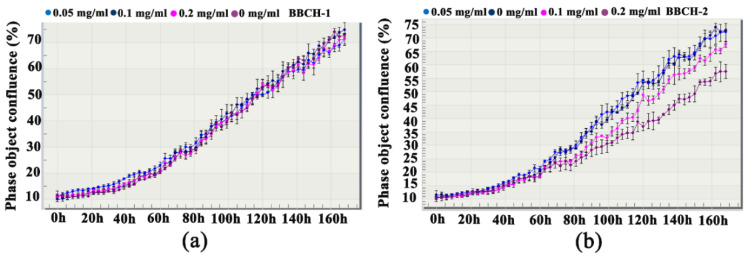
BBCH-1 and BBCH-2 fractions stimulated the proliferation of MC3T3-E1 cells. (**a**,**b**) MC3T3-E1 growth curves after treatment with BBCH-1 and BBCH-2, respectively. The y-axis is a label-free measure of cell confluence utilized by IncuCyte ZOOM live cell imaging system to evaluate cell proliferation. BBCH, bovine bone collagen hydrolysate.

BBCH was fractioned into two fractions, BBCH-1 (MW < 3000 Da) and BBCH-2 (MW > 3000 Da), by ultrafiltration. Stimulation of osteoblast proliferation by these two fractions was evaluated using the IncuCyte system [13]. BBCH-1 (0.05 mg/mL) promoted proliferation of MC3T3-E1 osteoblasts within 72 h (Figure 1a). Thus, BBCH-1 was subjected to further investigation.

### 2.2. Identification of Peptides in the BBCH-1 Fraction

The sequences of BBCH-1 fraction peptides that had greater osteoblast proliferation stimulation activity were identified using nano-HPLC-MS-MS BBCH-1 (Waters, Milford, MA, USA) analysis and a Bos Taurus databasedriven search via PEAKS Studio 6.0 software (Bioinformatics Solution Inc., Waterloo, ON, Canada). Twenty-nine peptides were identified as were their calculated molecular masses, peptide sequences, and additional information (Table 1). The identified peptide length ranged from 10 to 31 amino acids and their corresponding calculated molecular masses ranged from 967.4545 to 2785.3391 Da. Moreover, 27 of the identified peptides were derived from collagen type I and only two were derived from collagen type X.

### 2.3. Molecular Docking for Screening Peptides that Interact with EGFR

Prior to docking, a suitable algorithm needed to be selected according to the standard of generated poses and the value of root mean square deviation. We chose to use Libdock and CDOCKER, two classical algorithms in Discovery Studio 2017 software, and MOEdock, an algorithm in Molecular Operating Environment 2014. The principle of Libdock is a scoring function based on hotpot matching, which is able to easily provide a rapid virtual screening [14,15]. CDOCKER is an algorithm that uses a CHARMM force field [14]. MOEdock is another forcefield-based scoring function and is able to estimate the free energy of binding with the ligand from a given pose [16]. To select an appropriate algorithm from these three candidates, molecular docking analysis between EGF and EGFR was performed for each of the algorithms. The three algorithms produced four (Libdock), five (CDOCKER), and six (MOEdock) poses, respectively. The minimum root mean square deviation values for each of the three algorithms were 15.0634 (Libdock), 14.228 (CDOCKER), and 14.8449 (MOEdock), respectively (Table 2). In addition, the CDOCKER function was the most accurate of the assessed algorithms [13]. Therefore, CDOCKER was the most suitable algorithm for this investigation and was used in subsequent analyses. The “CDOCKER Energy” value (Table 1) indicates the affinity between peptides and EGFR and ranged from 270.659 to 46.9511. Of all analyzed peptides, three peptides had a “CDOCKER Energy” greater than 200 kcal/mol. These three peptides were HHGDQGAPGAVGPAGPRGPAGPSGPAGKDGR (deamidation), HHGDQGAPGAVGPAGPRGPAGPSGPAGK (deamidation), and GPAGANGDRGEAGPAGPAGPAGPR (deamidation). Five peptides shared the same amino acid sequence (GPAGPSGPAGK). Moreover, the only difference between HHGDQGAPGAVGPAGPRGPAGPSGPAGKDGR (deamidation) and HHGDQGAPGAVGPAGPRGPAGPSGPAGK (deamidation) was the amino acid sequence DGR, which was responsible for the minor differences in their “CDOCKER” values. HHGDQGAPGAVGPAGPRGPAGPSGPAGKDGR (deamidation), GPAGANGDRGEAGPAGPAGPAGPR (deamidation), and GPAGPSGPAGKDGR were chosen for further study.

### 2.4. In Vitro Effect of Synthesized Peptides on MC3T3-E1 Cell Proliferation

**Figure 2:**
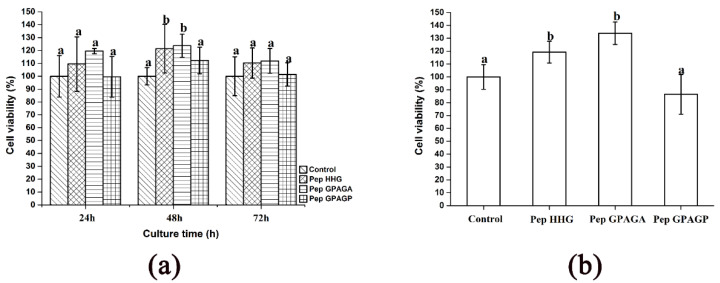
The relative cell viability of MC3T3-E1 osteoblast cells in response to Pep HHG, Pep GPAGA, and Pep GPAGP was measured using 3-(4,5-Dimethylthiazol-2-yl)-2,5-diphenyltetrazolium bromide (MTT) and CCK8 assays. (**a**) MTT assay results at 24, 48, and 72 h. (**b**) CCK8 assay results after 48 h. Different letters represent significant differences between groups, *p* < 0.05 Pep HHG: peptide HHGDQGAPGAVGPAGPRGPAGPSGPAGKDGR (Deamidation); Pep GPAGP: peptide GPAGANGDRGEAGPAGPAGPAGPR (Deamidation); Pep GPAGP: peptide GPAGPSGPAGKDGR.

The in vitro cell viability response of MC3T3-E1 cells to the synthesized peptides, at 0.05 mg/mL, was evaluated over 24–72 h using MTT and CCK8 assays. Peptide HHGDQGAPGAVGPAGPRGPAGPSGPAGKDGR (Deamidation) and peptide GPAGANGDRGEAGPAGPAGPAGPR (Deamidation) had the greatest influences on osteoblast MC3T3-E1 viability at 48 h (*p* < 0.05) and a peptide concentration of 0.05 mg/mL, leading to an increase in viability of 21.4% and 23.7%, respectively (Figure 2a,b). Unfortunately, the cell effect of the GPAGPSGPAGKDGR peptide on cell viability did not significantly differ from that of the control group.

### 2.5. Effects of Synthesized Peptides on Cell Cycle in MC3T3-E1 Cells

**Figure 3:**
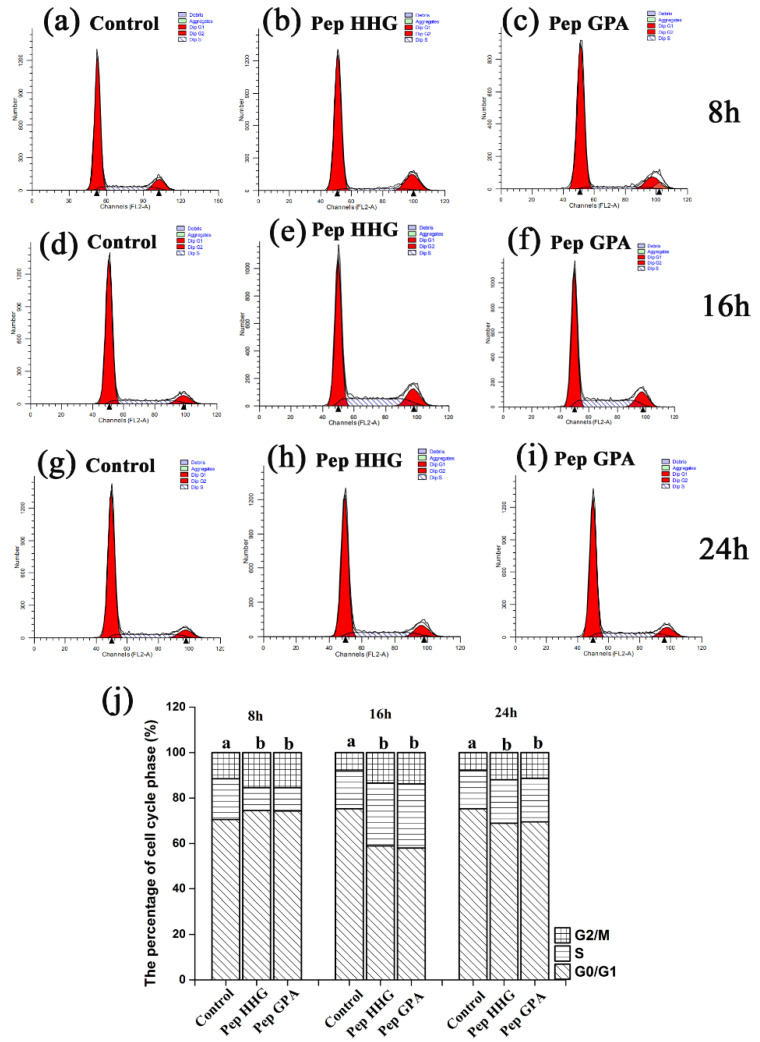
The cell cycle distribution of MC3T3-E1 osteoblasts cells after treatment with Pep HHG and Pep GPA and cultured for varying time periods assessed using flow cytometry (**a–i**). Cell cycle distribution of MC3T3-E1 osteoblasts in G0/G1, S, or G2/M phases (**j**). The different letters represent significant difference between groups, *p* < 0.05. (**a**–**c**) Control MC3T3-E1 osteoblasts and MC3T3-E1 osteoblasts treated with Pep HHG and Pep GPA for 8 h, respectively. (**d**–**f**) Control MC3T3-E1 osteoblasts and MC3T3-E1 osteoblasts treated with Pep HHG and Pep GPA for 16 h, respectively. (**g**–**i**) Control MC3T3-E1 osteoblasts and MC3T3-E1 osteoblasts treated with Pep HHG and Pep GPA for 24 h, respectively. Pep HHG: peptide HHGDQGAPGAVGPAGPRGPAGPSGPAGKDGR (Deamidation); Pep GPA: peptide GPAGANGDRGEAGPAGPAGPAGPR (Deamidation).

Peptide HHGDQGAPGAVGPAGPRGPAGPSGPAGKDGR (Deamidation) and peptide GPAGANGDRGEAGPAGPAGPAGPR (Deamidation), at 0.05 mg/mL, had the greatest effects on osteoblast proliferation. The effect of these peptides on the cell cycle was monitored in MC3T3-E1 cells cultured for 8, 16, and 24 h. Treatment with these peptides resulted in an increase in the percentage of S phase cells and a reduction in the percentage of G0/G1 phase cells (Figure 3).

### 2.6. Anti-Apoptotic Effects of the Synthesized Peptides

**Figure 4:**
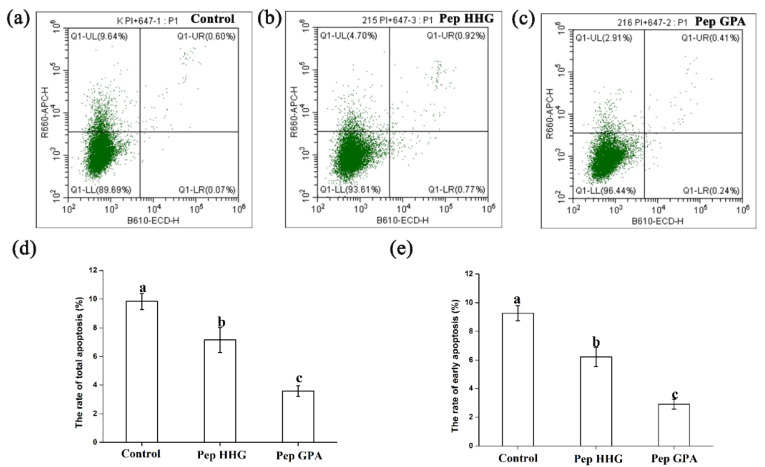
Apoptotic suppression in MC3T3-E1 osteoblast cells lines was measured using flow cytometry (**a**–**c**) and the calculated total apoptotic (**d**) and early apoptotic (**e**) rates. The different letters represent significance differences between groups, *p* < 0.05. (**a**–**c**) Control cells, cells treated with Pep HHG for 24 h, cells treated with Pep GPA for 24 h, respectively. Pep HHG: peptide HHGDQGAPGAVGPAGPRGPAGPSGPAGKDGR (Deamidation); Pep GPA: peptide GPAGANGDRGEAGPAGPAGPAGPR (Deamidation).

The effects of 24 h treatment with peptide HHGDQGAPGAVGPAGPRGPAGPSGPAGKDGR (Deamidation) and peptide GPAGANGDRGEAGPAGPAGPAGPR (Deamidation) on apoptosis in MC3T3-E1 cells stained with Annexin V (AV)-FITC (Fluoresceine Isothiocyanate) and propidium iodide (PI) were evaluated using flow cytometry. Apoptosis was inhibited by treatment with these two peptides at 0.05 mg/mL (Figure 4). The total rate of osteoblast MC3T3-E1 apoptosis decreased from 9.845% to 7.155% or 3.58% after treatment with peptide HHGDQGAPGAVGPAGPRGPAGPSGPAGKDGR (Deamidation) and peptide GPAGANGDRGEAGPAGPAGPAGPR (Deamidation), respectively. These results show that peptide GPAGANGDRGEAGPAGPAGPAGPR (Deamidation) had a greater influence on apoptosis than did peptide HHGDQGAPGAVGPAGPRGPAGPSGPAGKDGR (Deamidation). These results also show that these two peptides inhibit early apoptosis in MC3T3-E1 osteoblast cells. The rate of early apoptosis for peptide HHGDQGAPGAVGPAGPRGPAGPSGPAGKDGR (Deamidation) decreased from 9.265% to 6.205%, and for peptide GPAGANGDRGEAGPAGPAGPAGPR (Deamidation), the rate of early apoptosis decreased from 9.265% to 3.155%.

### 2.7. Molecular Docking between Synthesized Peptides and EGFR

**Figure 5:**
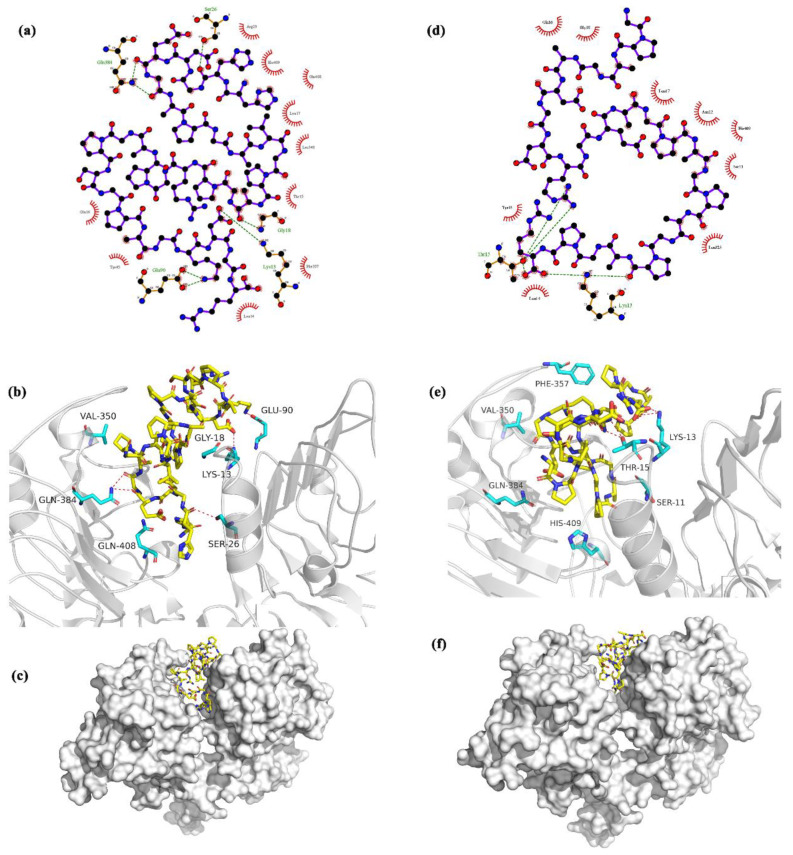
Molecular docking analysis between epidermal growth factor receptor (EGFR) (PDB: 1lVO) and Pep HHG and Pep GPA. (**a**) The 2D view of the Pep HHG binding model with EGFR. The peptide is shown in ball-stick mode in the middle of the figure. The green dotted lines depict the hydrogen bond with the EGFR amino acid sidechain. The red semicircle line represents the hydrophobic interaction between EGFR amino acid residues and the ligand peptide. (**b**) The 3D view of the peptide HHG binding model with the target. The carbon atoms of the ligand are in yellow, and the surrounding binding pocket residues are cyan. The receptor backbone is depicted as a light gray ribbon. The red dotted line shows the hydrogen bond between the ligand and receptor. (**c**) The surface overview of the peptide HHG binding model of the target. The carbon atoms of the ligand are in yellow, and the receptor surface is white. (**d**) The 2D view of the Pep GPA binding model with EGFR. The peptide is shown in ball-stick mode in the middle of the figure. The green dotted lines depict the hydrogen bond with the EGFR amino acid sidechain. The red semicircle line represents the hydrophobic interaction between EGFR amino acid residues and the ligand peptide. (**e**) The 3D view of peptide GPA binding model with EGFR. The carbon atoms of the ligand are in yellow, and the surrounding binding pocket residues are cyan. The receptor backbone is depicted as a light gray ribbon. The red dotted line shows the hydrogen bond between the ligand and receptor. (**f**) The surface overview of the peptide GPA and EGFR binding model. The carbon atoms of the ligand are in yellow, and the receptor surface is white. Pep HHG: peptide HHGDQGAPGAVGPAGPRGPAGPSGPAGKDGR (Deamidation); Pep GPA: peptide GPAGANGDRGEAGPAGPAGPAGPR (Deamidation).

The HHGDQGAPGAVGPAGPRGPAGPSGPAGKDGR (Deamidation) binding model showed that the peptide interacts with EGFR (Figure 5a–c). The Ser26, Gln384, Glu90, Lys13, and Gly18 receptor side chain formed hydrogen bonds with HHGDQGAPGAVGPAGPRGPAGPSGPAGKDGR (Deamidation). The peptide ligand also hydrophobically interacted with Arg29, His409, Gln408, Leu17, Leu348, Thr15, Phe357, Leu14, Tyr45, and Gln16 in the pocket of the receptor. The GPAGANGDRGEAGPAGPAGPAGPR (Deamidation) binding model showed that the peptide interacts with EGFR (Figure 5d–f). The Thr15 and Lys13 side chain in the receptor binding pocket formed hydrogen bonds with this peptide. The peptide ligand also hydrophobically interacted with Gln16, Gly18, Leu17, Asn12, His409, Ser11, Leu325, Leu14, and Tyr45 in the pocket of the receptor.

## 3. Discussion

In recent years, a variety of bioactivities for collagen peptides have been reported, such as antihypertensive [17], antioxidant [18], and antidiabetic actions [19], in addition to beneficial effects on bone. A great volume of evidence indicates that the bioactivity of collagen peptides is related to their molecular weight. For instance, squid skin collagen peptides with the lowest molecular weight (HSSG-III < 2000 Da) possess the most potent anti-ACE (angiotensin I-converting enzyme) bioactivity among the three fractions, namely, HSSG-I (6–10 kDa), HSSG-II (2–6 kDa), and HSSG-III (<2 Ka) [20]. *Dasidicus gigas*-derived collagen hydrolysates of low molecular weights, ranging from 500 to 1400 Da, show cytotoxic and antiproliferative activities in MCF-7 (human breast carcinoma) and U87 cell lines (glioma) [21]. Collagen peptides from squid skin that are between 1400 Da and 500 Da exert the highest antioxidant ability compared with those of greater mass [20]. Peptides from collagen hydrolysates of cobia skin that are below 700 Da significantly affect the antioxidant properties of these hydrolysates [22]. Two novel peptides with the highest calcium-chelating activity were isolated from pacific cod skincollagen hydrolysates and identified as GDKGESGEAGER and GEKGEGGHR [23]. Similarly, the beneficial effects of collagen on bone health are related to the molecular weight distribution. For example, pigskin collagen with molecular mass below 3000 Da was reported to inhibit bone loss caused by ovariectomizing and improve the microstructure of lumbar vertebrae [24]. Of all fractions, the lowest molecular mass collagen peptides from Crucian skin could bind more calcium and possessed a greater ability to improve calcium bioavailability in the retinoic acid-induced bone loss model [25]. In the present study, BBCH-I, with a molecular size of less than 3000 Da, exhibited stronger osteogenic bioactivity. In addition, this fraction contained numerous low molecular weight peptides (Table 1). Moreover, our previous study demonstrated that the fraction with MW 878 Da showed the most potent osteocalcin content increased activity among the four fractions, namely, MW 1370 Da, MW 2900 Da, and MW 7747 Da [26]. Therefore, there may exist a correlation between osteogenic activity and the molecular size of peptides.

In addition, the types of collagen appear to correlate with the beneficial effects of hydrolyzed collagen on bone health [27]. Type I collagen hydrolysates might exert more impacts on bone health than do other types of collagen hydrolysates because they are capable of providing the primary building blocks required for renewing bone collagen. This is because type I collagen, which plays a vital role in inducement of osteoblastic differentiation, calcification, and the supply of structure and elasticity for bone, is the primary element of bone [28]. Moreover, there are many osteogenic peptides derived from collagen type I including the novel osteogenic peptides, GPSGPAGKDGRIGQPG and GDRGETGPAGPAGPIGPV [29]. In the present study, the majority of identified peptides (Table 1) were from collagen type I. Therefore, our results support the contention that there is a potential correlation between osteogenic peptides and the parental proteins from which they are derived.

The amino acid compositions and peptide sequences of peptides may affect their bioactivity [30]. Peptides comprised of more hydrophobic amino acid residues may have stronger stimulation potencies on osteoblasts as they have the potential to form a greater number of hydrophobic interactions with EGFR, which has a close relationship with bone formation. Additionally, peptides that have a higher percentage of amino acid residues such as glutamic acid may also have greater potential osteogenic activity because of a greater number of possibilities to form hydrogen bonds with EGFR. These inferences are supported by our data (Figure 5). Our results reveal five hydrogen bonds and ten hydrophobic interactions between peptide HHGDQGAPGAVGPAGPRGPAGPSGPAGKDGR (Deamidation) and the EGFR receptor, and two hydrogen bonds and nine hydrophobic interactions between peptide GPAGANGDRGEAGPAGPAGPAGPR (Deamidation) and EGFR. Moreover, it has been suggested that hydrogen bonds and hydrophobic interactions contribute to stimulation potency in osteoblasts MC3T3-E1 cells [29].

Cell proliferation is an important phase of bone formation, and both bone formation and skeletal integrity can be affected by the osteoblast’s viability [31]. Moreover, bovine bone collagen peptides with higher levels of osteoblast proliferation stimulation can also promote mineralization, the final phase of bone formation [10]. In this study, we found that peptide HHGDQGAPGAVGPAGPRGPAGPSGPAGKDGR (Deamidation) and peptide GPAGANGDRGEAGPAGPAGPAGPR (Deamidation) displayed significant osteoblast proliferation stimulation bioactivity (*p* < 0.05). Therefore, these two peptides might be utilized as functional food for stimulating osteoblast proliferation to exert a beneficial effect on bone formation.

Cell proliferation can be adjusted via cell cycle phase alteration, which causes indirect effects on bone formation, including bone mineralization [32]. Collagen peptides can alter cell cycle distribution in B16F10 melanoma cells [33] and chum salmon skin collagen hydrolysates can alter the cell cycle progression [34]. Our results revealed that the proportion of cells in S phase was elevated and the proportion of cells in G0/G1 phase decreased significantly when treated with peptide HHGDQGAPGAVGPAGPRGPAGPSGPAGKDGR (Deamidation) and peptide GPAGANGDRGEAGPAGPAGPAGPR (Deamidation). These results suggest that these peptides can promote the proliferation of MC3T3-E1 osteoblast cells though alteration of cell cycle progression.

Apoptosis, a process that helps organisms remove unwanted cells to precisely control organ function and development, determines osteoblast lifespan in their final phase of formation, which is crucial for bone remodeling [35]. The antiapoptotic effects of collagen hydrolysates from chum salmon skin have been verified [34]. In this study, we found that peptide HHGDQGAPGAVGPAGPRGPAGPSGPAGKDGR (Deamidation) and peptide GPAGANGDRGEAGPAGPAGPAGPR (Deamidation) decreased apoptosis. These results indicate that these peptides might promote proliferation of MC3T3-E1 cells by preventing apoptosis.

The EGFR family includes four members: EGFR, erbB2, erbB3, and erbB4. These receptors can be activated via forming oligomers with ligands such as EGF. EGFR, a transmembrane glycoprotein, is frequently activated. The activation of the EGFR signaling pathway is an indicator of cell proliferation and inhibition of apoptosis [36,37]. In other words, the intro cellular signaling cascade, including cell proliferation survival and anti-apoptosis, occurs in response to ligand stimulation, namely, the EGFR signaling pathway is ligand-dependent [38]. In order to screen bioactive peptides from collagen hydrolysates, molecular analysis between EGFR with identified peptides is an efficient and economical way. In addition, molecular docking, a computational simulation method that is able to assist in predicting sites between receptor and ligand, has been used to better screen bioactive peptides with osteoblasts proliferation stimulation from protein hydrolysates [39]. For instance, Shi has successfully screened a peptide, namely ENLPEKADRDQYEL, with promoting proliferation proliferation of osteoblast activity using molecular docking analysis between EGFR and identified peptides from bovine lactoferrin hydrolysate [5]. In this study, peptide HHGDQGAPGAVGPAGPRGPAGPSGPAGKDGR (Deamidation) and peptide GPAGANGDRGEAGPAGPAGPAGPR (Deamidation) may activate EGFR signaling similarly to EGF because they interact with similar sites within the receptor, such as Phe 357 and Val 350. These results suggest that the underlying mechanisms of cell cycle alteration, promotion of cell proliferation, and prevention of apoptosis might be related to the EGFR signaling pathway. However, the mechanisms of the bioactivities observed for the proteins examined need to be further investigated and verified.

## 4. Materials and Methods

### 4.1. Materials

Bovine bone collagen hydrolysates (BBCHs) were prepared in our laboratory. Briefly, collagen was initially extracted from bovine bone, followed by enzymatic hydrolysis using papain and trysin in sequence to produce collagen hydrolysates. MC3T3-E1 osteoblasts and their growth media (Alpha modified Eagle’s minimum essential medium (with nucleosides), α-MEM-N medium) and media supplement (fetal bovine serum, FBS) were bought from the National Infrastructure Cell line Resource (Beijing, China). The 3-(4,5-Dimethylthiazol-2-yl)-2,5-diphenyltetrazolium bromide (MTT) and CCK8 kit were purchased from Solarbio Science and Technology Co., Ltd. (Beijing, China). All reagents used were of analytical grade.

### 4.2. BBCH Fractionation via Ultrafiltration

Using a Pellicon ultrafiltration system (Millipore, MA, USA) coupled with a 3000 Da molecular weight (MW) cut-off membrane, BBCH dissolved in distilled water was fractioned into two fractions: BBCH-1 (MW < 3000 Da) and BBCH-2 (MW > 3000 Da). The two fractions were then lyophilized and subjected to cell proliferation assay.

### 4.3. BBCH-1 and BBCH-2 Cell Proliferation Assays

MC3T3-E1 osteoblasts were seeded in a 96-well plate at a density of 750 cells/well in α-MEM-N medium containing 20% FBS. Cell proliferation assays were performed using an IncuCyte live cell analysis system (Essen Instruments, Ann Arbor, MI, USA). Confluence measurements were used to assess the proliferation stimulation bioactivity of BBCH-1 and BBCH-2 (at concentrations of 0.2, 0.1, and 0.05 mg/mL) in osteoblasts.

### 4.4. BBCH-1 Nano-HPLC-MS-MS Analysis

The fraction with the greatest bioactivity (osteoblasts proliferation activity) was dissolved in 10 μL distilled water containing 0.1% formic acid. Subsequently, the fraction was further purified using a nano Aquity UPLC system (Waters, Milford, MA, USA) and analyzed using quardrupole-Orbitrtrap mass spectrometry (Q-Exactive; Thermal Fisher Scientific, Bremen, Germany) coupled with an online nano-electrospray ion source. The sample of 4 μL was loaded onto the trap column (Thermal Scientific Acclaim PepMap C18, 100 μm × 2 cm) with a flow rate of 10 μL/min for 3 min and then purified onto the analytical column (Acclaim PepMap C18, 75 μm × 25 cm) using a linear gradient (5% B–30% B in 85 min, A: distilled water containing 0.1% of formic acid; B: Acetonitrile containing 0.1% of formic acid) at a flow rate of 300 nL/min with a constant column temperature of 45 °C. Electrospray voltage of 2.0 KV versus the inlet of the mass spectrometer was used. All MS/MS data were obtained through HPLC-MS_MS analysis and then searched against the Uniprot proteome Bos taurus database via PEAKS Studio 6.0 software (Bioinformatics Solution Inc., Waterloo, ON, Canada).

### 4.5. Molecular Docking

Molecular docking analysis between peptides with greater bioactivity and EGFR was performed using Discovery Studio (DS) software and Molecular Operating Environment (MOE) software (version 2014, WeiCompu Ltd., Beijing, China). The EGFR-EGF protein–ligand complex (PDB ID: 1IVO) crystal structure was obtained from the RCSB Protein Data Bank (https://www.rcsb.org) and served as the docking target and the template for comparing the docking mode peptides bound to EGFR [40]. CDOCKER, Libdock, and Moedock classical algorithms were used for the docking model. A suitable algorithm needed to be validated based on the standard of the root mean square deviation (RMSD) values and the number of generated poses. For receptor preparation in the DS software, removing water molecules, adding hydrogen, removing EGF ligands, and constructing the missing loop regions were performed by the Prepare Protein module. Additionally, the original position (EGF site) in the 1IVO complex served as the active site for docking simulation. For receptor prepared in the MOE software, the protonation and orientation of the hydrogens were optimized by LigX.

### 4.6. Peptide Synthesis

Peptides with greater osteogenic activity were synthesized using the solid-phase method (GL Biochem Ltd., Shanghai, China) based on the results of molecular docking analyses. These peptides were 96% pure and their sequences was validated using HPLC-MS-MS. The remaining 4% was TFA (tallow fatty acid) salt and some by products.

### 4.7. MTT Assay and CCK8 Assay for Synthesized Peptides

Cell medium containing 750 MC3T3-E1 cells (200 μL) was seeded into 96-well plates and cultured in a Stericycle CO_2_ incubator (Thermal fisher Scientific Inc., Waltham, MA, USA). After incubating for 24, 48, and 72 h, proliferation assays were performed using the MTT and CCK8 methods to evaluate the bioactivity of the synthesized peptides in stimulating osteoblast proliferation.

### 4.8. Cell Cycle Analysis

MC3T3-E1 osteoblast cells were seeded into six-well plates at a density of 3 × 10^5^ cells/well and treated with α-MEM-N medium containing synthesized peptides (0.05 mg/mL) for 8, 16, and 24 h. Cells not treated with peptides were used as the control. Harvested cells were subjected to cell cycle analysis using flow cytometry (FACS Calibur, Becton Dickson, San Jose, CA, USA).

### 4.9. Apoptosis Analysis

MC3T3-E1 cells seeded into six-well plates at a density of 3 × 10^5^ cells/well, either treated with synthesized peptides or untreated, were cultured for 24 h. Then, the FITC-annexin V/PI detection kit was used to analyze apoptosis.

### 4.10. Statistical Analysis

The statistically significant differences were calculated using SPSS software (version 20.0, SPSS Inc., Chicago, IL, USA). Significant differences between the mean values of three or more experiments were calculated using one-way analysis of variance. With LSD (L) (least-significant-difference) and S–N–K (Studen-Newman-Keuls) procedure, *p* < 0.05 was considered statistically significant.

## 5. Conclusions

Two novel peptides, HHGDQGAPGAVGPAGPRGPAGPSGPAGKDGR (Deamidation) and GPAGANGDRGEAGPAGPAGPAGPR (Deamidation), were identified based on their affinity with the EGFR receptor and their bioactivities. Additionally, our results show that these identified bioactivity peptides from bovine bone collagen hydrolysates function through the underlying mechanisms of anti-apoptosis, cell cycle alteration, and stimulation of proliferation, and might be related to the EGFR signaling pathway. Moreover, there might be a correlation between the bioactivities of peptides and their sequences, molecular sizes, and the types of collagen from which they are derived. Our results provide a foundation for the application of these peptides as functional components of functional gelatin products.

## Figures and Tables

**Table 1:** Molecular peptide profiles from the bovine bone collagen hydrolysate (BBCH)-1 fraction as identified by nano-HPLC-MS-MS.

Rank	Modification (s)	Peptide Sequence	Cdocker_Energy	Calculated Mass	Length (Amino Acids)	Parent Protein
1	Deamidation	HHGDQGAPGAVGPAGPRGPAGPSGPAGKDGR	270.659	2785.3391	31	Collagen alpha-2(I) chain
2	Deamidation	HHGDQGAPGAVGPAGPRGPAGPSGPAGK	226.104	2457.1897	28	Collagen alpha-2(I) chain
3	Deamidation	GPAGANGDRGEAGPAGPAGPAGPR	219.025	2056.9673	24	Collagen alpha-2(I) chain
4	Carbamylation	GAAGLPGPKGDRGDAGPK	177.177	1662.8438	18	Collagen alpha-1(I) chain
5	Deamidation	ANGDRGEAGPAGPAGPAGPR	162.153	1774.8346	20	Collagen alpha-2(I) chain
6	None	APGAVGPAGPRGPAGPSGPAGK	142.022	1824.9594	22	Collagen alpha-2(I) chain
7	Sulfation	GAPGAVGPAGPRGPAGPSGPAGK	133.665	1961.9376	23	Collagen alpha-2(I) chain
8	Acetylation	GARGEPGPAGLPGPPGER	126.109	1712.8594	18	Collagen alpha-1(I) chain
9	Sulfation	GPRGPAGPSGPAGKDGR	125.316	1612.7375	17	Collagen alpha-2(I) chain
10	None	KGDIGPAGLPGPR	112.306	1233.6829	13	Collagen alpha-1(X) chain
11	Sulphone	STGISVPGPMGPSGPR	107.425	1527.7351	16	Collagen alpha-1(I) chain
12	Hydroxylation	ARGPSGPQGPSGPPGPK	106.07	1558.7852	17	Collagen alpha-1(I) chain
13	Oxidation	STGISVPGPMGPSGPR	105.231	1511.7402	16	Collagen alpha-1(I) chain
14	Sulphone	TGISVPGPMGPSGPR	104.886	1440.7031	15	Collagen alpha-1(I) chain
15	Dihydroxy	STGISVPGPMGPSGPR	101.712	1527.7351	16	Collagen alpha-1(I) chain
16	Oxidation	TGISVPGPMGPSGPR	98.1569	1424.7081	15	Collagen alpha-1(I) chain
17	Dihydroxy	GFPGLPGPSGEPGK	94.1413	1327.6407	14	Collagen alpha-1(I) chain
18	Hydroxylation	ARGPSGPQGPSGPPGPK	92.3435	1558.7852	17	Collagen alpha-1(I) chain
19	None	GDIGPAGLPGPR	90.1347	1105.5879	12	Collagen alpha-1(X) chain
20	Sulphone	GISVPGPMGPSGPR	87.741	1339.6554	14	Collagen alpha-1(I) chain
21	Hydroxylation	RGPSGPQGPSGPPGPK	87.5512	1487.748	16	Collagen alpha-1(I) chain
22	Oxidation	GISVPGPMGPSGPR	87.3568	1323.6605	14	Collagen alpha-1(I) chain
23	Hydroxylation	GLTGPIGPPGPAG	84.569	1105.5768	13	Collagen alpha-1(I) chain
24	Hydroxylation	GLTGPIGPPGPAGA	81.6947	1176.6139	14	Collagen alpha-1(I) chain
25	Hydroxylation	GPSGPQGPSGPPGPK	78.866	1331.647	15	Collagen alpha-1(I) chain
26	None	AGPAGPAGPAGPR	77.6665	1074.557	13	Collagen alpha-2(I) chain
27	Dihydroxy	GISVPGPMGPSGPR	71.7777	1339.6554	14	Collagen alpha-1(I) chain
28	Oxidation	VPGPMGPSGPR	63.247	1066.5229	11	Collagen alpha-1(I) chain
29	Oxidation	PGPMGPSGPR	46.9511	967.4545	10	Collagen alpha-1(I) chain

Deamidation: the removal of an amid group.

**Table 2:** Molecular docking between EGF and EGFR using the Libdock, MOEdock, and CDOCKER algorithms.

Libdock			MOEdock			CDOCKER		
Pose	Reference	RMSD (Å)	Pose	Reference	RMSD (Å)	Pose	Reference	RMSD (Å)
1IVO 1	1IVO 4	15.0634	1IVO 1	1IVO 6	15.2038	1IVO 1	1IVO 5	12.9719
1IVO 2	1IVO 4	18.8533	1IVO 2	1IVO 6	15.3858	1IVO 2	1IVO 5	14.228
1IVO 3	1IVO 4	17.3093	1IVO 3	1IVO 6	25.5119	1IVO 3	1IVO 5	21.2551
1IVO 4	1IVO 4	0	1IVO 4	1IVO 6	14.8449	1IVO 4	1IVO 5	21.8973
−	−	−	1IVO 5	1IVO 6	24.1174	1IVO 5	1IVO 5	0
−	−	−	1IVO 6	1IVO 6	0	−	−	−

RMSD: root mean square deviation; EGF: epidermal growth factor; EGFR: epidermal growth factor receptor.
